# COX-2 structural analysis and docking studies with gallic acid structural analogues

**DOI:** 10.1186/2193-1801-1-58

**Published:** 2012-12-10

**Authors:** M Amaravani, Nirmal K Prasad, Vadde Ramakrishna

**Affiliations:** Department of Biotechnology & Bioinformatics, Yogi Vemana University, Kadapa, 516 003 A,P INDIA

**Keywords:** Cyclooxygenase, COX-2, Gallic acid, Indian gooseberry, Docking studies

## Abstract

*Emblica officinalis* is an ayurvedic herbal plant. The compounds isolated from this plant have good inhibitory effects against cyclooxygenase-2 (COX-2), among them gallic acid (GA) has the highest inhibitory effect. COX-2 (1.14.99.1) is an oxidoreductase having a role in prostaglandin biosynthesis, inflammatory responses and in cardiovascular events. COX-2 has gained special focus on research since past few decades. The sequence and structural studies reveals *Mus musculus* COX-2 shares the common conserved sequence and structural pattern with human COX-2. Molecular modeling and docking analysis with gallic acid and their structural analogues showed that 2-[(2E,4E)-hexa-2,4-dienyl]-3,4,5-trihydroxybenzoic acid, (3,4,5-trihydroxybenzoyl) 3,4,5-trihydroxybenzoate and 3-hydroxy-4-sulfooxybenzoic acid are more interactive and binding strongly than gallic acid at active site. Hence these three compounds should be considered as strong inhibitors for COX-2.

## Introduction

COX-1 and COX-2 are two distinct isoforms of cyclooxygenase, and plays a vital role in conversion of arachidonic acid to prostaglandins (Lipsky et al. [1998]; Vane et al. [1998]). Prostaglandins (PGs) are involved in various pathophysiological processes like inflammatory responses, carcinogenesis and in cardiovascular events. COX-2 is not detectible in most normal tissues, but is induced by proinflammatory cytokines, growth factors and carcinogens, implying a role for COX-2 in both inflammation and control of cell growth (Subbaramaiah et al. [1996]). In inflammatory tissues such as rheumatoidal synovium expression of COX-2 is up regulated and produce prostaglandin precursors which ultimately converted in to prostaglandins (Prasit et al. [1999]). The recent studies on selective inhibition of COX-2 caused suppression of inflammation and azoxymethane-induced colon cancer have shown the importance of COX-2 as a target for anti-inflammatory and anticancer therapy (Dannhardt and Kiefer, [2001]; Subhashini et al. [2004]; Amaravani et al. [2006]). Taken together, these data strongly suggest that suppressing levels of COX-2 will be an effective strategy for inhibiting inflammation and carcinogenesis.

Non-steroidal anti-inflammatory drugs (NSAIDs) are effective against inflammation and are observed to inhibit PG biosynthesis. NSAIDs inhibit both isoforms of cyclooxygenases (COX), but they are also associated with well-known side effects such as gastrointestinal side effects and renal function suppression (Herschman, [1996]). It is known that selective COX-2 inhibitors can provide anti-inflammatory agents devoid of the undesirable effects associated with classical non-selective NSAIDs (DeWitt, [1999]). As a consequence, increasing interest has been devoted to the synthesis of inhibitors of COX-2 by means of modification of well-known non-selective agents. Apart from selective and non-selective inhibitors, many natural products have also been identified as COX-2 inhibitors (Zhang et al. [1999]). As part of the search for natural anti-inflammatory agents from medicinal plants, *Emblica officinalis* extracts showed good medicinal values towards inflammation. Gallic acid (GA) is a naturally occurring polyhydroxyphenolic compound and an excellent free radical scavenger to inhibit COX isoforms (Madlener et al. [2007]; Pal et al. [2010]; Reddy et al. [2010]). Presence of high levels of gallic acid in *Emblica officinalis* gives a special status and medicinal value for treating inflammatory diseases (Ramakrishna et al. [2011]).

The present work focuses on the structural analysis of COX-2, interaction studies with gallic acid at active site and screening of gallic acid structural analogues. COX-2 active site analysis and molecular docking analysis enabled us to find better inhibitors as compared to gallic acid. These interaction studies are very useful to understand the mechanism of COX-2 catalyzed enzymatic reactions as well as the role of bioactive compounds interaction with active site residues. The approach is applicable in engineering 3D structures of enzymatic models, and studying interactions of active site residues with ligands (Nirmal et al. [2011a]).

## Material and methods

### Secondary structural analysis

Human COX-2 protein and its structural homologue protein sequences were retrieved from the NCBI protein database (http://www.ncbi.nlm.nih.gov). Pair wise sequence alignment of sequences was generated by Clustal W 2.0 (http://www.ebi.ac.uk/Tools/clustalw2/index.html) and analyzed to map the secondary structural conservation and variations. Secondary structural analysis was carried out by using Bioedit 7.0 (Hall, [1999]) and Discovery Studio Viewer (http://www.accelrys.com).

### COX-2 Homology Modeling and optimization

To build the COX-2 homology model, a BLASTp algorithm against Protein Data Bank (PDB) was used to carry out the sequence homology searches. Crystal structure of *Mus musculus* cyclooxygenase 2 (PDB ID: 1PXX) was taken as a template to build homology model. The Modeller 9v7 program (Sali and Blundell, [1993]) was employed to generate the 3D models of COX-2. The model with high score was validated by the Procheck (Laskowski et al. [1993]), VADAR (Willard et al. [2003]) and ProSA (Wiederstein and Sippl, [2007]). Further the model was refined by energy minimization. The energy minimization was performed using the NAMD package (Phillips et al. [2005]). The optimized model was subjected to quality assessment with respect to its geometry and energy and then subjected to molecular docking. Ramachandran plot was utilized for geometric evaluation. ProSA program was employed to evaluate the quality of model and examine the energy of residue–residue interactions using a distance-based pair potential. The gallic acid and its structural analogue molecules downloaded from Pubchem database of NCBI (Wang et al. [2009]), and converted to 3D structure with VEGA ZZ software (Pedretti et al. [2004]). These molecules were geometrically optimized for further use in docking. C alpha and back bone atoms root mean square deviation (RMSD) of template and COX-2 model was calculated by magic fit program (Guex and Peitsch, [1997]).

### Model energy minimization and molecular dynamics

3D structure refinement of COX-2 was carried out using energy minimization and molecular dynamics. It was performed using Nano Molecular Dynamics (NAMD 2.6). The simulations and energy minimization were carried out in 50,000 step minimization of the designed side chains and solvent to remove bad contacts. Minimum switching distance of 8.0 Å and a cut off of 12.0 Å for Vander Walls interactions was used, pair list of the non-bonded interactions was recalculated every 20 steps with a pair list distance of 13.5 Å. The resultant energy minimized protein models were used for the active site identification and for docking with substrates.

### Active site analysis

The substrate accessible pockets and active sites of COX-2 were identified by computed atlas of surface topography of proteins (CASTp) calculation (Dundas et al. [2006]) and CCDC GOLD (Jones et al. [1997]; Verdonk et al. [2003]). To test the accessibility of the pockets were tested by docking with randomly selected inhibitor molecules. The identified pockets were analyzed for amino acid cluster groups based on the solvent exposed active site atoms and bonding capacity of the polar groups.

### Docking analysis and inhibitor screening

Gallic acid and its structural analogues are obtained from Pubchem database of NCBI and converted into 3D structures with VEGA ZZ software. The docking was carried out at the binding sites by CCDC’s GOLD (genetic optimization for ligand docking). One-hundred genetic algorithm (GA) runs were performed for each compound, and 10 ligand bumps were allowed in an attempt to account for mutual ligand/target fit. The binding region for the docking study was defined as a 10 Å radius sphere centered on the active site. For each of the GA run a maximum number of 100,000 operations were performed on a population of 100 individuals with a selection pressure of 1.1. The number of islands was set to 5 with a niche size of 2. The weights of crossover, mutation, and migration were set to 95, 95, and 10 respectively. The scoring function Gold Score implemented in GOLD was used to rank the docking positions of the molecules, which were clustered together when differing by more than 2 Å RMSD (Phogat et al. [2010]; Nirmal et al. [2011b]). The best ranking clusters for each of the molecules were selected. Hydrogen bonds, bond lengths and close contacts between enzyme active site and ligand atoms were analyzed.

## Results and discussion

### Secondary structural features

**Figure 1:**
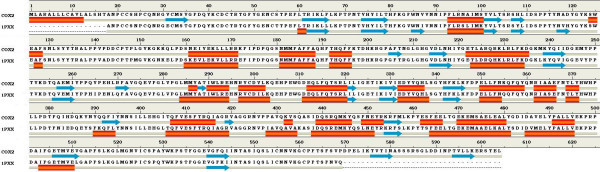
**Secondary structural comparison of human COX-2 and template** .

Comparative secondary structural analysis of COX-2 with template reveals that the secondary structural elements were well conserved. The secondary structural comparison of COX-2 was presented in Figure 1. Secondary structure of the COX-2 showing same pattern as compared to template secondary structure except few small stretches of beta sheets (2 to 3 amino acids) but this can be ignored.

### COX-2 model

**Figure 2:**
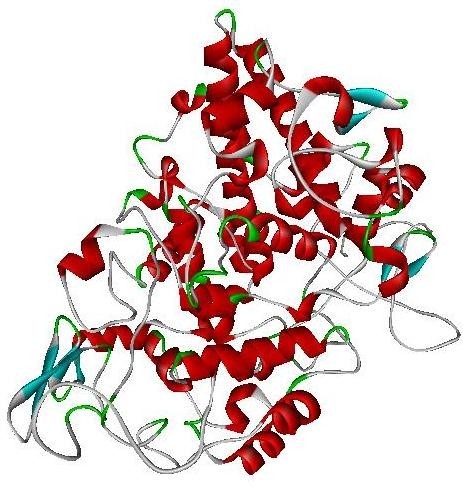
**Final 3- D model of COX-2 (A) and superimposition with the template (B)** .

**Figure 3:**
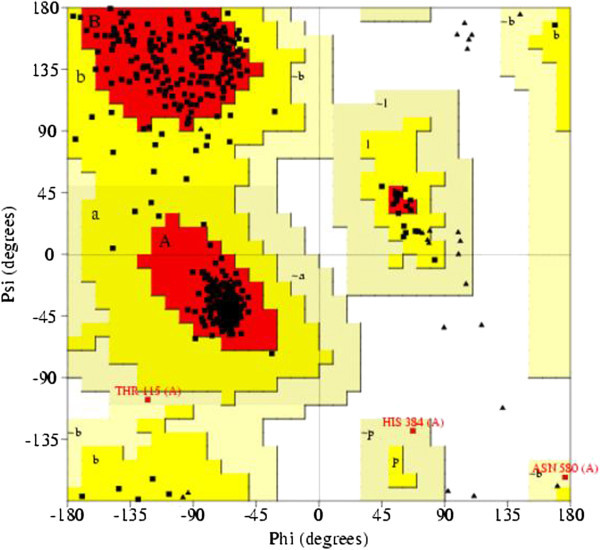
**COX-2 homology model Ramachandran plot** .

The COX-2 is a 604 amino acids protein. Crystal structures of COX-2 from different species have already been determined and available in PDB. Among them, *Mus musculus* cyclooxigenase 2 (PDB ID: 1PXX) showed the highest sequence identity (87%) with COX-2. Practically, at this level of sequence identity, it is good enough to use 1PXX as a template, in order to obtain high quality alignment for the structure prediction by homology modeling. COX-2 homology (A) and superimposed pose with Template (B) was shown in Figure 2. The geometry of the final model of COX-2 was evaluated with Ramachandran’s plot calculations computed with the PROCHECK program. This result revealed 91.8% of the residues were in the core region, 7.6% residues in the allowed regions and 0.6% in generously allowed region. COX-2 Ramachandran plot was depicted in Figure 3. The PROSA analysis of the model showed maximum residues to have negative interaction energy with very few residues displayed positive interaction energy and the overall interaction energy of the model was −7.69 kcal/mol, which is quite similar to the template Z score.

Cα atoms and back bone atoms RMSD of the model and template was 0.35 Å. The mean residue volume and total packing volume of the model are 153.9 Å^3^ and 92962.6 Å^3^ respectively. VADAR analysis of the model showed, the mean helix phi, psi and omega angles are − 65.1, − 40.4 and −178.3 respectively, which is promising residue packing when compared to the crystal, structure information. Hence, the final model which proved to be well validated in terms of geometry and energy profiles suggests that the model is good enough to be an initial point for our next stage of molecular docking studies.

### Active site composition

**Figure 4:**
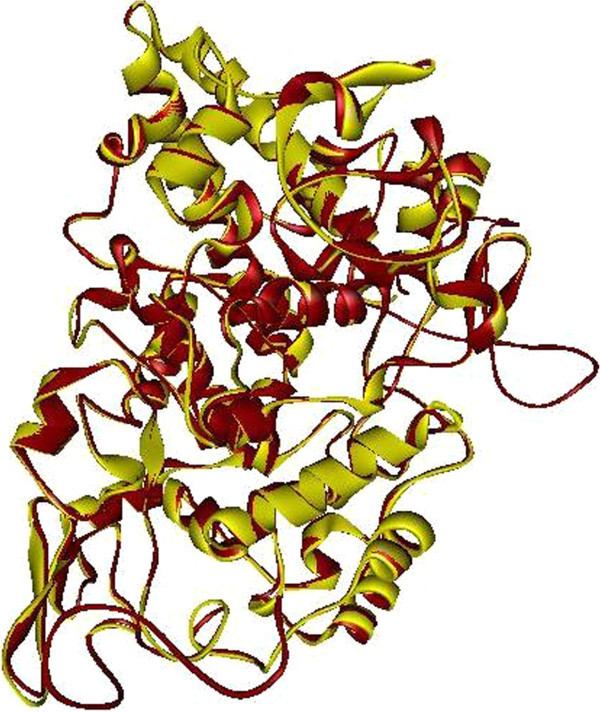
**Surface representation of active site pocket** .

After the final homology model was built, the possible ligand-binding site of COX-2 was searched by CASTp calculation and CCDC GOLD. Non-steroidal anti-inflammatory drug (NSAID) binding site was selected for docking studies. The volume and area of active site are 5331.2 Å^3^ and 1651.6 Å^2^ respectively. The active site accommodate by 25 amino acids i.e., ALA185, PHE186, PHE187, ALA188, GLN189, HIS190, THR192, HIS193, GLN194, PHE196, THR198, ASN368, LEU370, TYR371, HIS372, TRP373, HIS374, LEU376, LEU377, VAL433, SER437, GLN440, TYR490, LEU493 and LEU494. There were 5 hydrogen donor groups present in the active site. COX-2 active site was shown in Figure 4. The comparison of the overall folding and the structure of active site between COX-2 and the template protein reveal a high structural homology. Active site composition features were depicted in Table 1.

**Table 1:** **Properties of COX-2 active site residue composition and accessible atoms**

S.No	Residue	No. of Hydrogen donors	Atoms
1	ALA185	-	-
2	PHE186	-	-
3	PHE187	-	-
4	ALA188	-	-
5	GLN189	-	-
6	HIS190	-	-
7	THR192	1	HG1
8	HIS193	-	-
9	GLN194	-	-
10	PHE196	-	-
11	THR198	-	-
12	ASN368	1	2HD2
13	LEU370	-	-
14	TYR371	-	-
15	HIS372	-	-
16	TRP373	1	H
17	HIS374	1	HE2
18	LEU376	-	-
19	LEU377	-	-
20	VAL433	-	-
21	SER437	-	-
22	GLN440	1	1HE2
23	TYR490	-	-
24	LEU493	-	-
25	LEU494	-	-

### COX-2 interaction analysis with inhibitors

**Figure 5:**
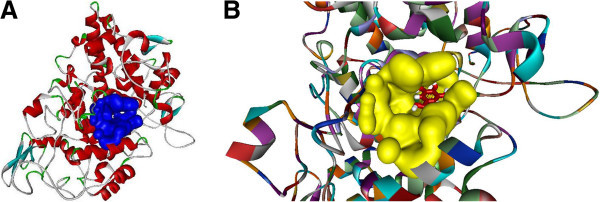
**Docked conformations of (A) Gallic acid and (B) 2-[(2E,4E)-hexa-2,4-dienyl]-3,4,5-trihydroxybenzoic acid in the active site pocket** .

Initial screening of gallic acid structural analogues was done by CCDC GOLD docking. There were 59 gallic acid structural analogues are screened. All the screened gallic structural analogues were accessibleand downloaded from the library (http://www.ioib.in/products/GASAL). This initial screening studies revealed 2-[(2E,4E)-hexa-2,4-dienyl]-3,4,5-trihydroxybenzoic acid, (3,4,5-trihydroxybenzoyl) 3,4,5-trihydroxybenzoate, 3-hydroxy-4-sulfooxybenzoic acid, 3,4-dihydroxy-2-sulfooxybenzoic acid, prop-2-enyl 3,4,5-trihydroxybenzoate, 4-hydroxybutyl 3,4,5-trihydroxybenzoate, 3-hydroxypropyl 3,4,5-trihydroxybenzoate, bis(3,4,5-trihydroxyphenyl)methanone and 1-(3,4,5-trihydroxyphenyl)pentan-1-one molecules having high affinity at active site and binding firmly. Further docking analysis of the screened inhibitors revealed 2-[(2E,4E)-hexa-2,4-dienyl]-3,4,5-trihydroxybenzoic acid, (3,4,5-trihydroxybenzoyl) 3,4,5-trihydroxybenzoate and 3-hydroxy-4-sulfooxybenzoic acid are producing high Gold fitness score which shows high binding affinity at active site. The docking conformations of COX-2 with screened inhibitors were shown in Figure 5. The Gold Score of all interactions reveals that, among all the ligands, 2-[(2E,4E)-hexa-2,4-dienyl]-3,4,5-trihydroxybenzoic acid exhibits the highest fitness score of 45.40. COX-2 docking statistics were depicted in Table 2.

**Table 2:** **Docking statistics**

S.No	Ligand	Gold score	H bond atoms	H Bond length (Å)
1	3,4,5-trihydroxybenzoic acid (Gallic acid)	28.2848	ALA185:O-H15	1.745
			ALA188:O-H16	2.692
2	2-[(2E,4E)-hexa-2,4-dienyl]-3,4,5-trihydroxybenzoic acid	45.4076	THR192:HG1-O3	2.099
			HIS372:ND1-H24	1.832
3	(3,4,5-trihydroxybenzoyl) 3,4,5-trihydroxybenzoate	42.7486	ALA185:O-H32	1.441
			THR192:HG1-O5	1.363
			ASN368:1HD2-O7	2.161
			ASN368:2HD2-O3	2.071
			HIS374:H-O8	2.298
4	3-hydroxy-4-sulfooxybenzoic acid	41.8640	THR192:HG1-O7	2.448
			ASN368:O-H2O	1.942
			ASN368:2HD2-O6	1.849
			HIS372:2ND1-H19	2.399
			TRP373:O-H21	2.561
5	3,4-dihydroxy-2-sulfooxybenzoic acid	40.5943	THR192:HG1-O3	2.407
			ASN368:O-O4	2.410
			ASN368:1HD2-O4	2.525
			TYR371:O-H19	2.219
			THR373:H-O2	2.456
			THR373:H-O5	2.271
6	prop-2-enyl 3,4,5-trihydroxybenzoate	40.2194	THR192:HG1-O2	2.127
			HIS372:ND1-H21	1.586
7	4-hydroxybutyl 3,4,5-trihydroxybenzoate	39.9954	THR192:OG1-H29	1.839
			THR192:HG1-O3	2.322
			THR192:HG1-06	2.009
			ASN368:2HD2-O4	2.546
8	3-hydroxypropyl 3,4,5-trihydroxybenzoate	39.9464	ALA185:O-H28	1.954
			THR192:OG1-H25	2.275
			THR192:HG1-O2	2.305
9	bis(3,4,5-trihydroxyphenyl)methanone	39.0007	ALA185:O-H30	1.847
			THR192:OG1-H25	2.124
			THR192:HG1-O2	2.181
			ASN368:O-H27	1.490
			ASN368:1HD2-O6	2.024
10	1-(3,4,5-trihydroxyphenyl)pentan-1-one	38.8825	TRP192:HG1-O2	1.995
			HIS372:ND1-H28	1.451
			TRP373:H-O1	2.563

## Conclusion

COX-2 plays a prime role in the prostaglandins biosynthesis pathway as it provides prostaglandin H_2,_ which is precursor for the formation of all other prostaglandins. Homology model of COX-2 showed 91.8% of the residues were in the core region, 7.6% residues in the allowed regions and 0.6% in generously allowed region of Ramchandran plot, suggesting the modeled COX-2 structure was reliable for the docking studies. The active site analysis showed 25 residues are present at surface accessible region of COX-2 active site. Top ten ranked gallic acid structural analogues on docking reveals that the 2-[(2E,4E)-hexa-2,4-dienyl]-3,4,5-trihydroxybenzoic acid has more affinity at active site than others. This information has potential implications to understand the mechanism of COX-2 related enzymatic inhibition reactions, and also applicable in the prediction of more effective inhibitors and engineering 3D structures of other enzymes as well.
